# Clinical phenotype and prognostic determinants of spontaneous pneumomediastinum in anti-MDA5 antibody-positive dermatomyositis

**DOI:** 10.3389/fimmu.2026.1852637

**Published:** 2026-05-29

**Authors:** Xiaoying Wang, Yusheng Zhang, Lijun Liu, Wei Li, Xiaojun Liu, Yan Wang

**Affiliations:** 1 Department of Rheumatology and Immunology, The First Affiliated Hospital of Zhengzhou University, Zhengzhou, Henan, China; 2 Department of Respiratory and Critical Care Medicine, The First Affiliated Hospital of Zhengzhou University, Zhengzhou, Henan, China

**Keywords:** anti-MDA5 antibody, cyclophosphamide, dermatomyositis, mortality risk, spontaneous pneumomediastinum

## Abstract

**Introduction:**

To identify the clinical phenotype and prognostic determinants in patients with anti-MDA5 antibody-positive dermatomyositis (MDA5+DM) with spontaneous pneumomediastinum (SPM).

**Methods:**

We retrospectively analyzed 150 MDA5+DM patients (50 with SPM and 100 without). Clinical, laboratory, and therapeutic profiles were analyzed. Multivariable logistic regression was used to identify factors associated with survival in the SPM cohort.

**Results:**

The SPM group had a significantly higher 6−month mortality than the non−SPM group (54% vs. 16%, P<0.001). Patients with SPM were older and less frequently had arthralgia, and exhibited a hyper−inflammatory phenotype [elevated CRP, serum ferritin (SF), LDH, neutrophil-to-lymphocyte ratio (NLR), KL-6], severe lymphopenia, and higher infection rates. Prior calcineurin inhibitor (CNI) use was more common in the non−SPM group (85% vs. 62%, P = 0.001). Within the SPM group, non-survivors had shorter disease duration and higher inflammatory markers. Multivariable analysis identified longer pre-SPM disease duration (OR 0.479, 95% CI 0.266–0.863) and prior cyclophosphamide (CTX) therapy (OR 0.034, 95% CI 0.003–0.405) as factors independently associated with improved survival (both P<0.05).

**Conclusion:**

Older MDA5+DM patients with a hyper-inflammatory phenotype and fewer arthralgias are at increased risk for SPM, whereas prior CNI use may be protective. In SPM patients, elevated inflammatory markers predict poor survival, while longer pre-SPM disease duration and prior CTX therapy are associated with improved survival, suggesting early aggressive immunosuppression may improve outcomes.

## 1 Introduction

Dermatomyositis (DM) is an autoimmune disorder characterized by immune−mediated myopathy and pathognomonic cutaneous lesions. Interstitial lung disease (ILD) constitutes a frequent and severe complication, representing a leading cause of mortality in this population (1). The recognition of myositis−specific autoantibodies has enabled stratification into distinct clinical phenotypes. Among these, anti−melanoma differentiation−associated gene 5 (anti−MDA5) antibodies delineate a subset strongly associated with peripheral lymphopenia, ulcerative skin lesions, rapidly progressive ILD (RP−ILD), and high mortality rates (2, 3).

Spontaneous pneumomediastinum (SPM) is a rare but life−threatening complication in anti−MDA5−positive DM (MDA5+DM). Its occurrence often signals rapid clinical deterioration, frequently culminating in acute respiratory failure and poor prognosis (4, 5). Consequently, the early detection of high-risk patients and prompt therapeutic measures are essential for enhancing clinical outcomes (6). The objective of this study was to delineate the clinical characteristics of MDA5+DM patients who develop SPM, outline its unique manifestations, and determine prognostic factors. These insights could facilitate early risk assessment and inform clinical decision-making, potentially improving survival rates in this severe complication.

## 2 Materials and methods

### 2.1 Study population

A retrospective cohort study was conducted among patients with MDA5+DM who developed SPM and were admitted to The First Affiliated Hospital of Zhengzhou University between January 2019 and August 2025. For each SPM patient, two contemporaneous MDA5+DM patients without SPM were randomly selected from the same study period at a 1:2 ratio. No additional matching on age, sex, or other variables was performed. Inclusion criteria were (1): diagnosis of DM according to the Bohan and Peter criteria (7, 8) or clinically amyopathic dermatomyositis (CADM) as defined by Sontheimer (9) (2); age ≥18 years; and (3) serum positivity for anti-MDA5 antibody confirmed by standardized immunoassay. Exclusion criteria included (1): coexistence of other well-defined autoimmune diseases (2); presence of malignancies or other conditions mimicking myositis; and (3) incomplete clinical or laboratory data. SPM was diagnosed based on radiological confirmation of pneumomediastinum via high-resolution computed tomography (HRCT). For SPM patients, all clinical and laboratory data were collected at the time of SPM diagnosis; for non-SPM patients, data were collected at the corresponding matched time point. The study protocol was approved by the Ethics Committee of The First Affiliated Hospital of Zhengzhou University (Approval No: 2022-KY-0427).

### 2.2 Data collection

Comprehensive clinical data were systematically collected for all eligible MDA5+DM patients at the time of SPM diagnosis (or the matched time point for non-SPM patients). Demographic characteristics, clinical manifestations (documented as ever present prior to or at the time of SPM diagnosis), and detailed treatment records were obtained. Laboratory parameters included complete blood count, liver and renal function profiles, muscle enzymes (creatine kinase [CK], lactate dehydrogenase [LDH]), and inflammatory markers (serum ferritin [SF], C−reactive protein [CRP], erythrocyte sedimentation rate [ESR]). Imaging assessments, particularly HRCT of the chest, and infection status were documented. The NLR was calculated as the absolute neutrophil count divided by the absolute lymphocyte count. Prior treatment was defined as any exposure to the agent for at least 2 weeks before the date of SPM diagnosis (or the matched time point for non-SPM patients).

### 2.3 Statistical analysis

Statistical analyses were performed using SPSS software (version 27.0, IBM Corp., Armonk, NY, USA). Continuous variables with normal distribution were expressed as mean ± standard deviation and compared using the independent samples t-test. Non-normally distributed data were presented as median (interquartile range, IQR) and compared using the Mann-Whitney U test. Categorical variables were described as frequencies (percentages) and compared using the Chi-square test or Fisher’s exact test, as appropriate. Binary logistic regression analysis was employed to identify risk factors associated with mortality in the SPM group. A two-sided P-value<0.05 was considered statistically significant.

## 3 Results

### 3.1 Higher mortality and distinct hyper−inflammatory phenotype in the SPM group

A total of 150 patients were included in the final analysis, comprising 50 in the SPM group and 100 in the non−SPM group. The SPM group had a substantially higher 6−month mortality rate (54% vs. 16%, P<0.001) compared to the non−SPM group.

Demographic characteristics, clinical manifestations, laboratory parameters, and prior treatment profiles were compared between the two groups (**Table 1**). Patients in the SPM group were significantly older and presented with a lower frequency of arthralgia compared to the non−SPM group (all P < 0.001). In terms of hematological and inflammatory parameters, the SPM group exhibited significantly higher white blood cell (WBC) counts, neutrophil counts, and NLR (all P < 0.05). Additionally, levels of CRP, SF, and LDH were significantly elevated in the SPM group, alongside higher KL−6 levels and the positivity rate for anti−Ro52 antibodies (all P < 0.05). In contrast, CK levels were significantly lower in the SPM group (P < 0.05). Anti−MDA5 antibody titers did not differ significantly between the groups (P = 0.475).

**Table 1:** Baseline demographic and laboratory characteristics of MDA5+DM patients in SPM and non-SPM group.

Characteristic	SPM group (N = 50)	Non-SPM group (N = 100)	*P*
Demographic characteristics			
Male, n (%)	23 (46)	33 (33)	0.132
Age at onset, years	55 (49-64)	47 (39-52)	<0.001*
Disease duration, mons	3 (2-6)	2 (1-4)	0.155
Clinical manifestations			
Hoarseness, n (%)	5 (10)	3 (3)	0.280
Rash, n (%)	45 (90)	89 (89)	0.985
Fever, n (%)	30 (60)	46 (46)	0.119
Arthralgia, n (%)	16 (32)	62 (62)	<0.001*
Laboratory findings			
WBC, ×10^9^/L	6.6 (5.0-8.9)	5.2 (3.9-7.0)	0.002*
N, ×10^9^/L	5.5 (4.0-7.6)	3.8 (2.8-5.4)	<0.001*
Lym, ×10^9^/L	0.5 (0.3-0.8)	0.8 (0.5-1.1)	0.001*
NLR	10.1 (6.4-18.6)	4.8 (3.0-8.1)	<0.001*
CRP, mg/L	12 (3-28)	3 (1-12)	0.001*
SF, ng/mL	1681 (771-2826)	693 (253-1234)	<0.001*
LDH, U/L	396 (307-582)	326 (267-438)	0.002*
CK, U/L	38 (27-75)	59 (34-114)	0.007*
KL-6, U/mL	1659 (1115-1998)	982 (581-1559)	<0.001*
Anti-Ro52 (+), n (%)	36 (72)	46 (46)	0.003*
MDA5, U/mL	173 (140-196)	179 (158-200)	0.475
**Infection**, n (%)	33 (66)	22 (22)	<0.001*
Fungal, n (%)	27 (54)	16 (16)	<0.001*
Bacterial, n (%)	15 (30)	10 (10)	0.002*
Viral, n (%)	10 (20)	13 (13)	0.273
Treatment before SPM onset			
CTX, n (%)	25 (50)	48 (48)	0.861
CNI, n (%)	31 (62)	85 (85)	0.001*
JAKi, n (%)	12 (24)	33 (33)	0.241
IVIG, n (%)	38 (76)	72 (72)	0.668
TCZ/RTX, n (%)	6 (12)	3 (3)	0.168
Antifibrosis, n (%)	10 (20)	25 (25)	0.475

SPM, Spontaneous pneumomediastinum; WBC, white blood cell; N, neutrophil; Lym, lymphocyte; NLR, neutrophil-to-lymphocyte ratio; CRP, C−reactive protein; SF, serum ferritin; CK, creatine kinase; CTX, cyclophosphamide; CNI, calcineurin inhibitors; JAKi, Janus kinase inhibitors; IVIG, intravenous immunoglobulin; TCZ, tocilizumab; RTX, rituximab. Values are presented as number (%) or median (interquartile range). All P-values were evaluated by the chi-squared test, Fisher’s exact test, Student’s t test or Mann-Whitney’s U test, as appropriate. *P < 0.05.

Bold values indicate statistically significant P values (P < 0.05).

Lymphocyte subset analysis revealed that the SPM group had significantly reduced absolute counts of total lymphocytes, T cells, B cells, and CD4^+^ T cells, compared to the non−SPM group (**Supplementary Table 1**, all P < 0.05). The overall incidence of infection was significantly higher in the SPM group (P < 0.05), with both fungal and bacterial infections occurring more frequently (all P < 0.05). However, the prevalence of viral infections was comparable between the two groups (P > 0.05).

Regarding immunosuppressive therapies before SPM onset, the use of calcineurin inhibitors (CNI) was significantly more frequent in the non-SPM group (85% vs. 62%, P = 0.001). No significant differences were observed between the groups in the prior use of cyclophosphamide (CTX), JAK inhibitors (JAKi), intravenous immunoglobulin (IVIG), tocilizumab (TCZ) or rituximab (RTX), or antifibrotic agents (all P > 0.05).

### 3.2 Non−survivors exhibit elevated inflammatory and tissue−damage markers within the SPM group

Among the 50 MDA5+DM patients with SPM, 27 (54%) died during the six−month follow−up period. Compared to survivors, non−survivors presented with a significantly shorter disease duration at the time of SPM diagnosis and were less likely to have ever experienced arthralgia (all P < 0.05).

**Figure 1:**
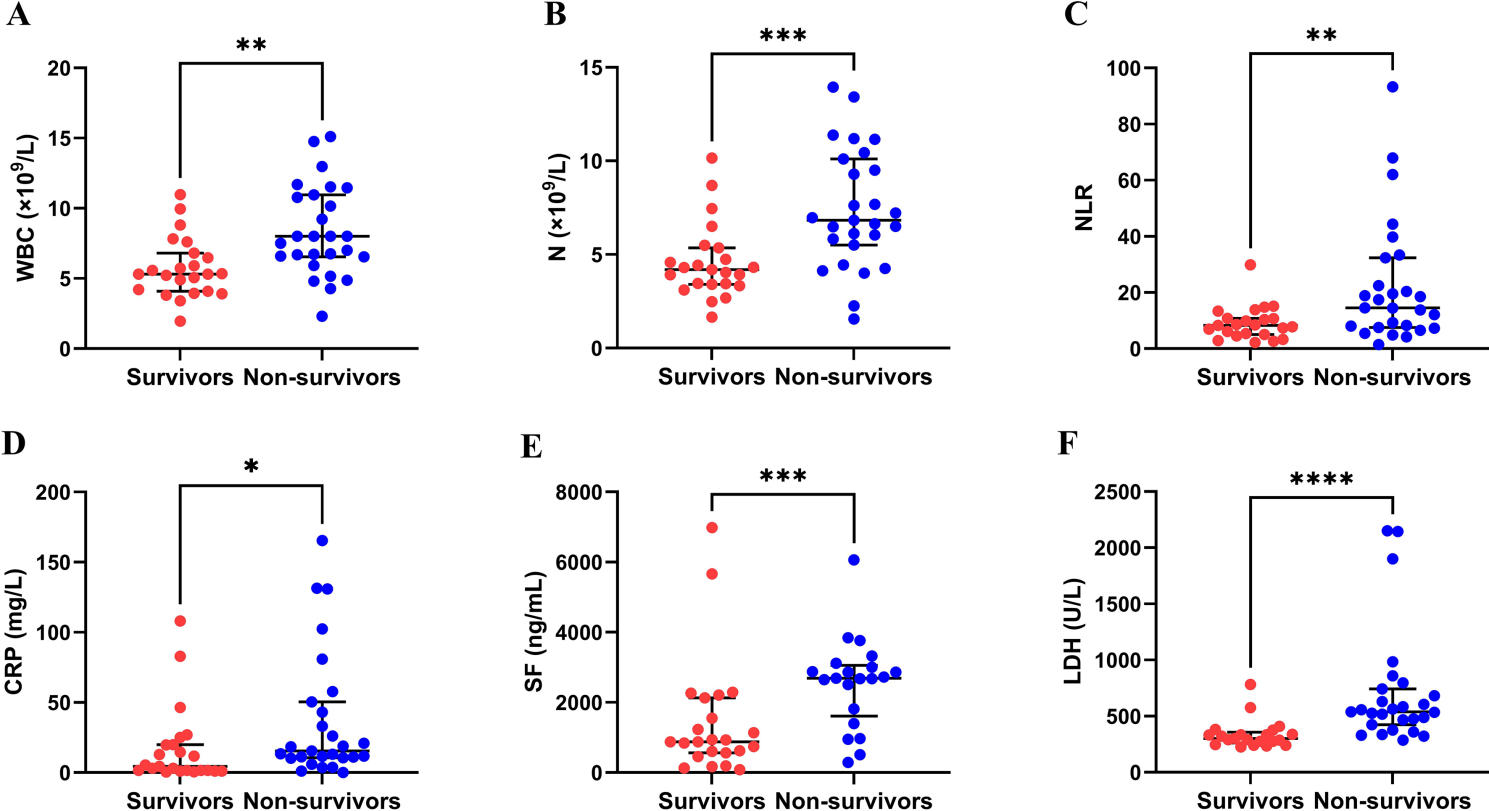
Comparison of laboratory parameters and clinical features between survivors and non-survivors in MDA5+DM patients with SPM. NLR, neutrophil-to-lymphocyte ratio; SF, serum ferritin. Data are presented as median (interquartile range). *P < 0.05, **P < 0.01, ***P < 0.001, ****P < 0.0001 (Mann-Whitney U test).

Non−survivors also exhibited significantly elevated levels of systemic inflammatory and tissue−damage markers, including WBC count, neutrophil count, NLR, CRP, SF, and LDH (**Figure 1**, all P < 0.05). No significant differences were observed in serum KL−6 levels, anti−Ro52 positivity rate, or anti−MDA5 antibody titers between survivors and non−survivors. Additionally, the prevalence of documented infection at SPM diagnosis was comparable between the two groups (**Table 2**).

**Table 2:** Baseline demographic and laboratory characteristics of survivors and non-survivors in MDA5+DM patients with SPM.

Characteristic	Survivors (n=23)	Non-survivors (n=27)	*P*
Demographic characteristics			
Male, n (%)	11 (47.8)	12 (44.4)	0.811
Age at onset, years	56 (51-61)	53 (49-65)	0.953
Disease duration, mons	6 (3-12)	2 (1-3)	<0.001*
Clinical manifestations			
Hoarseness, n (%)	2 (8.7)	2 (7.4)	1.000
Rash, n (%)	22 (95.7)	23 (85.2)	0.449
Fever, n (%)	15 (65.2)	15 (55.6)	0.487
Arthralgia, n (%)	11 (47.8)	5 (18.5)	0.027*
Laboratory findings			
CK, U/L	33 (25-45)	56 (31-86)	0.011*
KL-6, U/mL	1564 (1391-1974)	1688 (973-2111)	0.754
Anti-Ro52 (+), n (%)	14 (60.9)	22 (81.5)	0.106
MDA5, U/mL	167 (123-178)	179 (147-225)	0.089
Infection, n (%)	15 (65.2)	18 (66.7)	0.914
Treatment before SPM onset			
CTX, n (%)	18 (78.3)	7 (25.9)	<0.001*
CNI, n (%)	18 (78.3)	13 (48.1)	0.029*
JAKi, n (%)	6 (26.1)	6 (22.2)	0.750
IVIG, n (%)	19 (82.6)	19 (70.4)	0.313
TCZ/RTX, n (%)	3 (13.0)	3 (11.1)	1.000
Antifibrosis, n (%)	4 (17.4)	6 (22.2)	0.943

CK, creatine kinase; CTX, cyclophosphamide; CNI, calcineurin inhibitors; IVIG, intravenous immunoglobulin; RTX, rituximab. Values are presented as number (%) or median (interquartile range). All P-values were evaluated by comparing survivors and non-survivors using the chi-squared test, Fisher’s exact test, Student’s t test or Mann-Whitney’s U test, as appropriate. *P < 0.05.

Bold values indicate statistically significant P values (P < 0.05).

Regarding prior treatment exposure, survivors were significantly more likely to have received CTX (78.3% vs. 25.9%, P < 0.001) or CNI (78.3% vs. 48.1%, P = 0.029) before the diagnosis of SPM compared to non−survivors, with no significant differences observed between the groups in the prior use of JAKi, IVIG, TCZ, RTX, or antifibrotic agents (all P > 0.05) (**Table 2**).

### 3.3 Longer disease duration and prior CTX use are independently associated with improved survival in SPM patients

**Figure 2:**
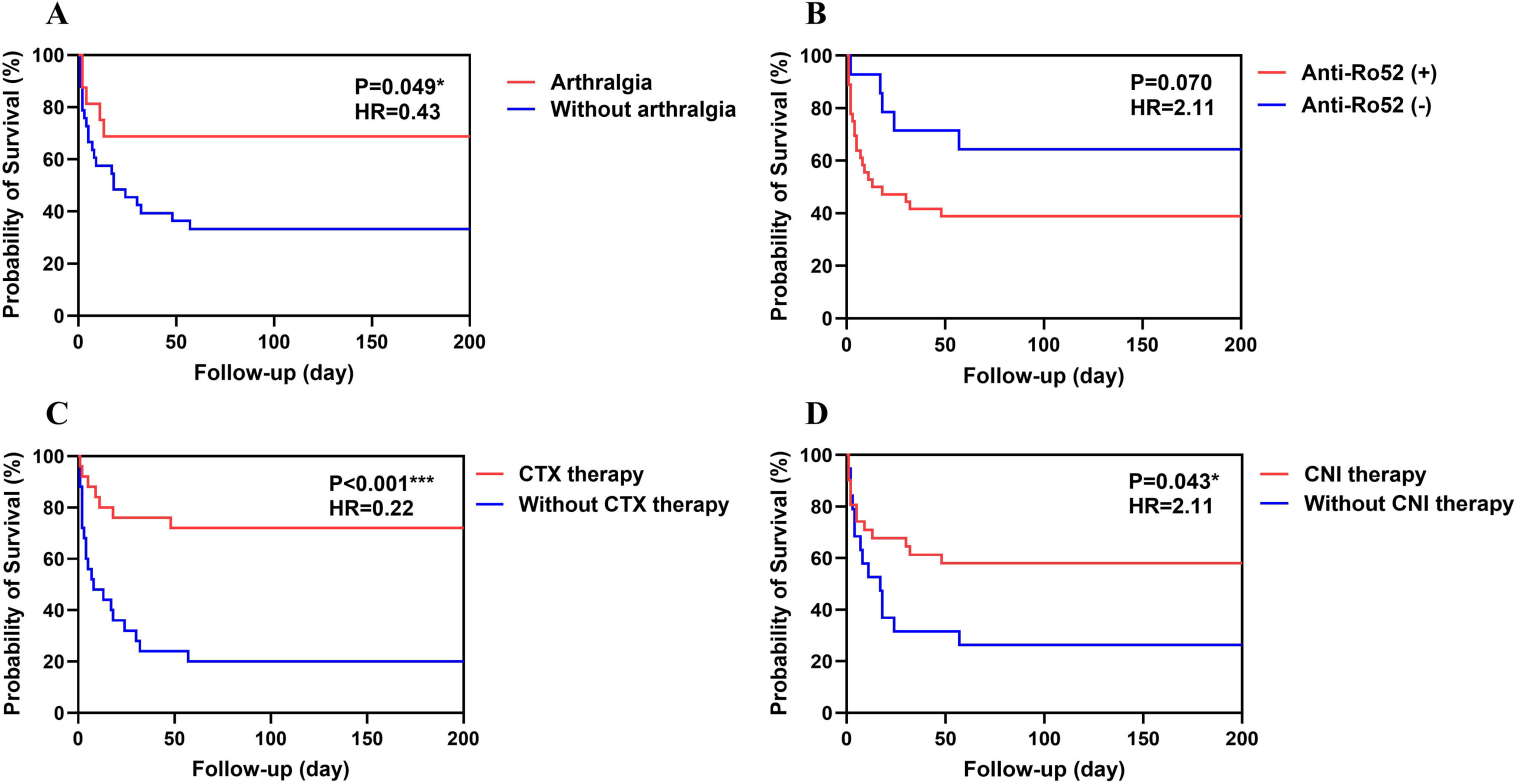
Kaplan-Meier survival curves of MDA5+DM patients stratified by clinical and treatment factors. **(A)** SPM patients with and without arthralgia; **(B)** SPM patients with anti-Ro52 (+) and anti-Ro52 (-); **(C)** SPM patients with and without cyclophosphamide (CTX) therapy; **(D)** SPM patients with and without calcineurin inhibitors (CNI) therapy. Cumulative survival rates were estimated by the Kaplan-Meier method. Differences in survival between groups were compared using the log-rank test. HR, hazard ratio; *P < 0.05, ***P < 0.001.

To identify independent prognostic factors for mortality in patients with SPM, logistic regression analysis was performed. Univariable analysis revealed that longer disease duration, presence of arthralgia, as well as prior treatment with CTX or CNI, were associated with improved survival. Kaplan-Meier survival curves for arthralgia, anti-Ro52 antibody status, prior CTX therapy, and prior CNI therapy are presented in **Figure 2**.

**Figure 3:**
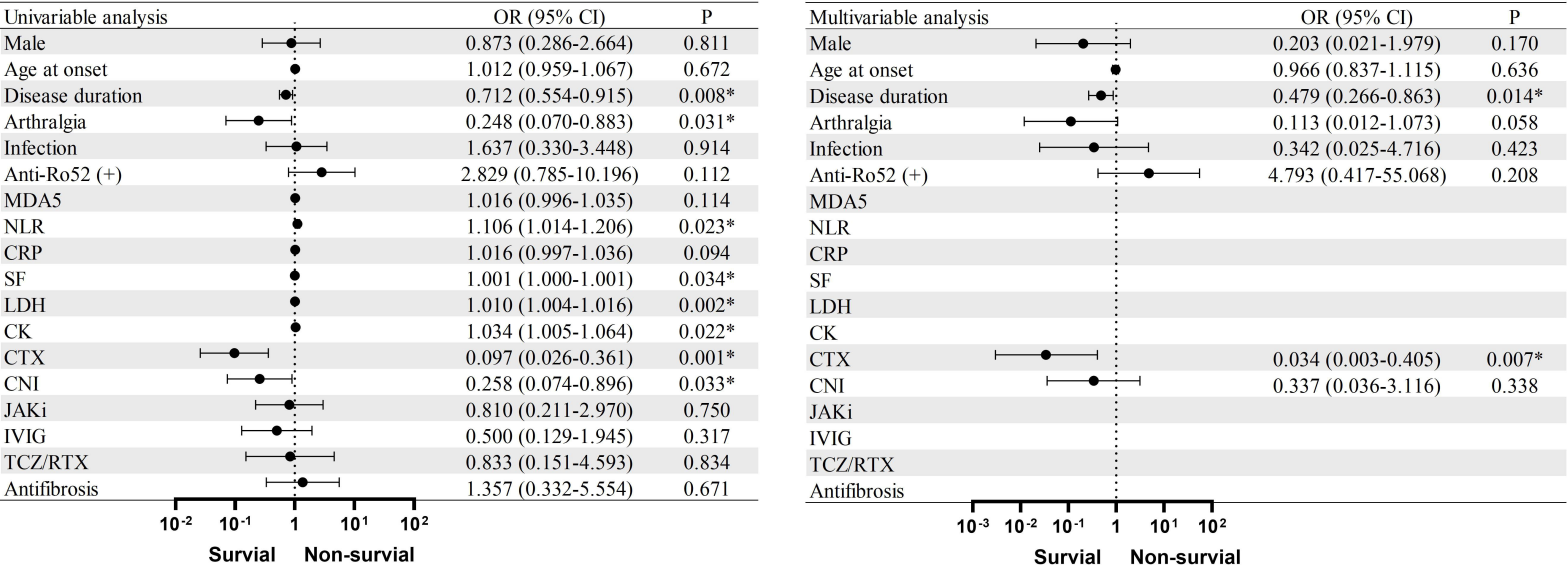
Univariable and multivariable Logistic regression analyses for factors associated with survival in MDA5+DM patients with SPM. The forest plot shows Odds ratios (ORs) and 95% confidence intervals (CIs) for each variable in univariate (left) and multivariable (right) analyses. Statistically significant associations are denoted with an asterisk (*P < 0.05). NLR, neutrophil-to-lymphocyte ratio; SF, serum ferritin; CTX, cyclophosphamide; CNI, calcineurin inhibitors; JAKi, Janus kinase inhibitors; IVIG, intravenous immunoglobulin; TCZ, tocilizumab; RTX, rituximab.

In contrast, elevated levels of systemic inflammatory and tissue−damage markers, including NLR, CRP, SF, LDH, and CK, were associated with increased mortality. Due to event number constraints and collinearity among inflammatory markers (NLR, CRP, SF, LDH, CK), the multivariable model instead adjusted for clinically important covariates including age, sex, disease duration, arthralgia, infection, anti−Ro52, prior CTX, and prior CNI. After multivariable adjustment for potential confounders, longer disease duration (OR 0.479, 95% CI 0.266–0.863; P = 0.014) and prior CTX therapy (OR 0.034, 95% CI 0.003–0.405; P = 0.007) remained independently associated with improved survival (**Figure 3**).

## 4 Discussion

In this study, SPM in anti−MDA5+DM patients was associated with a strikingly high 6−month mortality of 54%. Patients who developed SPM exhibited a hyper−inflammatory phenotype characterized by elevated CRP, SF, LDH, and NLR, along with severe lymphopenia and a higher burden of concurrent infections. Among SPM patients, longer pre−SPM disease duration and prior CTX therapy were independently associated with improved survival, whereas elevated inflammatory markers predicted poor outcomes in univariable analyses.

Anti-MDA5+DM is a distinct subtype of idiopathic inflammatory myopathy, often complicated by rapidly progressive interstitial lung disease (3, 10). Spontaneous pneumomediastinum (SPM) in this population is a particularly ominous sign, frequently heralding respiratory failure and high mortality (11, 12).

The clinical phenotype observed in our SPM cohort—encompassing advanced age, a marked hyper-inflammatory state (elevated CRP, SF, LDH), severe lymphopenia (impacting both T and B cell lineages), and a substantial load of concurrent infections—reinforces the concept of immune dysregulation and exuberant tissue injury (13, 14). This constellation aligns with a pathogenic model wherein extensive release of inflammatory mediators and lymphocyte trafficking into pulmonary tissues compromise both structural integrity and host defense mechanisms (15, 16).

In contrast, individuals without SPM demonstrated a reduced systemic inflammatory burden and an increased prevalence of arthralgia. This musculoskeletal-predominant phenotype may represent a distinct disease subset associated with better outcomes, characterized by less vasculopathy or rapidly progressive lung involvement (17). In these patients, articular manifestations often precede or coincide with interstitial lung disease (18, 19), allowing for earlier diagnosis and intervention—a concept further supported by the longer pre-SPM disease duration observed in our survivors.

A particularly intriguing and novel finding from our study is the potential protective association of prior CNI use against SPM development. While some observational studies have suggested a possible link between pre-existing immunosuppression and altered risk of SPM, the specific protective role of CNI has not been clearly established (20, 21). Our observation should therefore be interpreted with caution and may reflect a modulatory effect of early, targeted T-cell inhibition on the alveolar-capillary integrity or the initial hyper-inflammatory cascade predisposing to SPM, a hypothesis that merits prospective validation.

Within the SPM group, multivariable analysis identified longer pre-SPM disease duration and prior CTX therapy as independent protective factors against mortality. The association between shorter disease duration and poor survival aligns with recent large-cohort studies by Jin Q et al. (5), which identified early-onset SPM (within 3 months of ILD diagnosis) as an independent predictor of mortality (HR 1.59).

Regarding treatment, the strong protective association between CTX and improved survival suggests that early aggressive immunosuppression may be beneficial in high-risk patients. Jin Q et al. recently demonstrated that intensification of immunosuppressive therapy after SPM onset, defined as an increase in glucocorticoid dosage or the addition of immunosuppressive agents following SPM diagnosis, was associated with poor outcome (22). This finding contrasts with the protective association of pre-SPM CTX exposure observed in our cohort, suggesting that the impact of immunosuppression is critically time-dependent. Although early aggressive therapy may prevent or mitigate subsequent complications, once SPM is established with ongoing structural lung damage, further enhancing immunosuppression might no longer be beneficial and could increase the risk of infection or delayed tissue healing. Notably, although CNI use was associated with improved survival in univariable analysis (log-rank P = 0.043), this association was not independent after adjustment, likely reflecting confounding by concomitant CTX use or by less severe baseline disease in CNI-treated individuals. This clinical distinction is significant: CNI may assist in preventing SPM, but CTX, rather than CNI, is associated with improved survival after SPM develops.

Although elevated NLR, CRP, SF, and LDH emerged as potent predictors of mortality in univariable analyses, they could not be confirmed as independent factors in multivariable models due to statistical limitations from the small number of events. (n=27). This limitation does not reduce their clinical importance; instead, it highlights that mortality in SPM patients is influenced by a strong systemic inflammatory response, as indicated by these markers. The fact that these markers could not be added to the model without causing instability suggests that they are closely intertwined with the existing predictors or with each other, a finding consistent with the concept of a unified hyper-inflammatory phenotype. Future larger studies are needed to disentangle their relative contributions.

Interestingly, although our study identified a higher rate of infections in SPM patients, aligning with Jin J et al. (22) and Jin Q et al. (5) who linked CMV/fungal infections to SPM risk, we did not observe a survival difference based on infection status within the SPM subgroup. This may reflect the overwhelming impact of the hyper-inflammatory state itself on mortality, a factor potentially more dominant in our cohort’s high-risk patients. Indeed, non-survivors exhibited significantly elevated levels of systemic inflammatory and tissue damage markers (NLR, CRP, SF, LDH, CK), reinforcing that their mortality is more related to ongoing inflammation rather than to infection itself. However, the widespread empirical use of broad-spectrum antimicrobials in our cohort may have attenuated detectable differences in infection-related mortality between survivors and non-survivors.

There are several limitations in this study. First, the single-center retrospective design is difficult to eliminate selection bias or unmeasured confounding factors. Confounding by indication is particularly relevant: patients who received CTX or CNIs before SPM may have differed in baseline severity or prodromal duration, influencing both treatment and outcomes. Second, due to the limited number of outcome events (n=27), we were unable to perform a fully adjusted multivariable analysis incorporating all significant univariable predictors, particularly the inflammatory markers such as NLR, CRP, SF, and LDH. Specifically, the addition of any single inflammatory markers to the base model resulted in non-convergence, reflecting model saturation or collinearity. This statistical limitation may hinder the search for other independent prognostic factors and needs to be addressed in larger prospective cohort studies. Third, the temporal relationship between immunosuppression and SPM was not fully quantified; only exposure information before SPM was obtained, without detailed data on exposure time, duration, or cumulative dose. Consequently, we could not assess potential dose-response relationships or determine the minimum effective exposure required for clinical benefit. Despite these caveats, the strong association between CTX and survival, alongside consistent inflammatory biomarker signals, offers clinically meaningful insights that warrant prospective multicenter validation. Forth, although we excluded patients with incomplete data, we cannot rule out survivor bias: patients who died very early might have had fewer laboratory measurements and were thus more likely to be excluded. This could have attenuated the observed associations between inflammatory markers and mortality.

## 5 Conclusion

This study identifies a high-risk phenotype for SPM development in MDA5+DM, characterized by older age, hyper-inflammation, and absence of arthralgia, while prior CNI use may confer protection against this devastating complication. Once SPM occurs, mortality is exceedingly high, with elevated NLR, CRP, SF, and LDH serving as potential prognostic markers. Critically, longer pre-SPM disease duration and prior CTX therapy were independently associated with lower mortality, supporting early aggressive immunosuppression with CTX in high-risk patients. Prospective multicenter validation is warranted to refine risk-stratified strategies.

## Data Availability

The raw data supporting the conclusions of this article will be made available by the authors, without undue reservation.
