# Supplementary figures and images for: Clinical phenotype and prognostic determinants of spontaneous pneumomediastinum in anti-MDA5 antibody-positive dermatomyositis
Source: Front Immunol. 2026 May 29;17:1852637. doi: 10.3389/fimmu.2026.1852637 (PMC13260135; doi:10.3389/fimmu.2026.1852637)

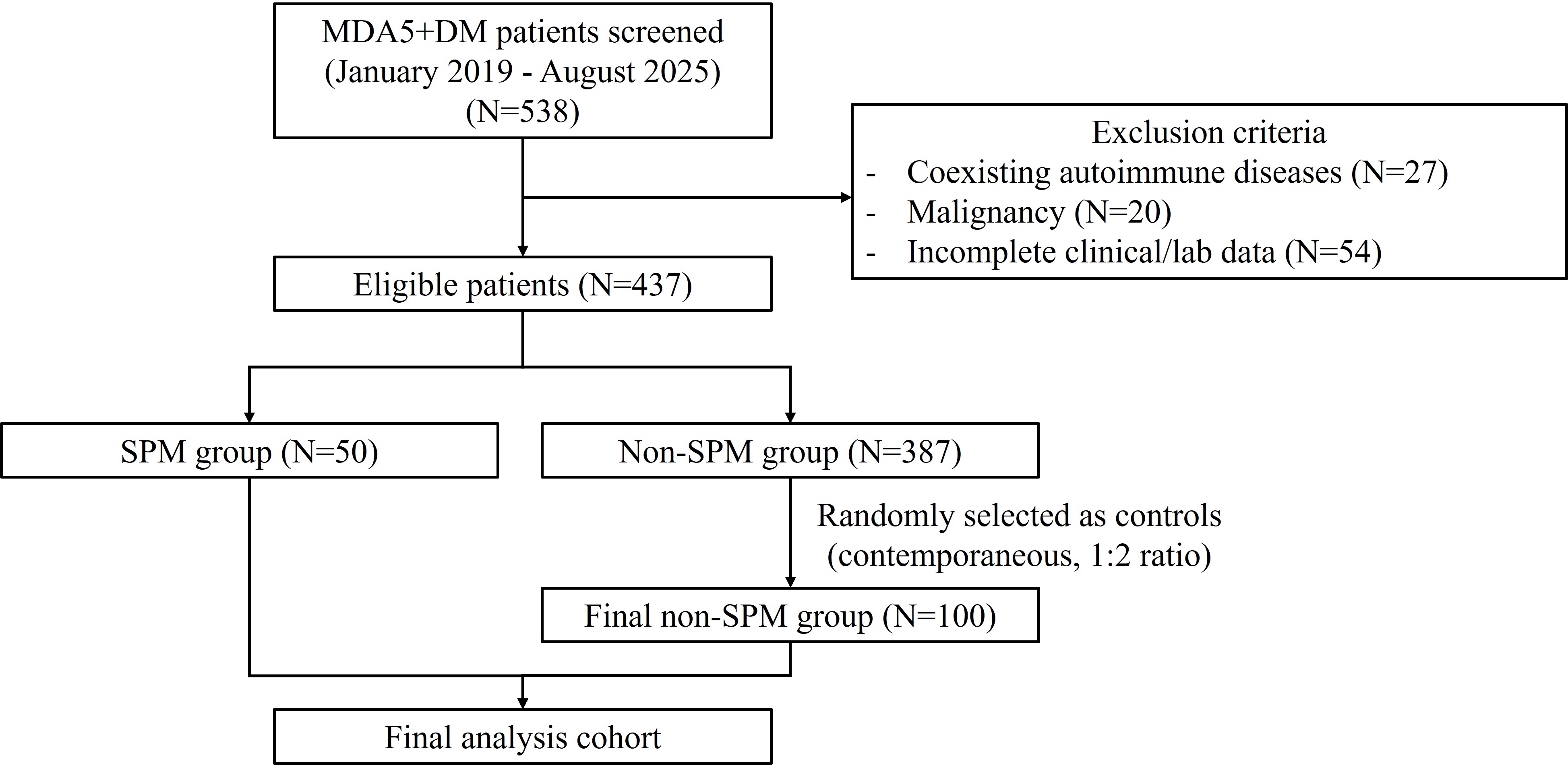

Supplement: Supplementary file 1 [file Image1.jpeg]
