# Supplementary material for: Clinical phenotype and prognostic determinants of spontaneous pneumomediastinum in anti-MDA5 antibody-positive dermatomyositis
Source: Front Immunol. 2026 May 29;17:1852637. doi: 10.3389/fimmu.2026.1852637 (PMC13260135; doi:10.3389/fimmu.2026.1852637)
Supplement: Supplementary file 2 [file Table1.docx]

**Supplementary Table S1 Lymphocyte subpopulation of MDA5+DM patients in SPM and non-SPM group**

|  | **SPM group**  **(N=50)** | **non-SPM group**  **(N=100)** | ***P*** |
| --- | --- | --- | --- |
| **Lymphocyte subpopulation** |  |  |  |
| B, /uL | 40 (20-115) | 121 (35-213) | **<0.001*** |
| T, /µL | 336 (232-501) | 526 (295-727) | **0.006*** |
| CD4+, /µL | 182 (125-259) | 333 (159-466) | **0.001*** |
| CD8+, /µL | 119 (86-219) | 153 (107-284) | 0.060 |
| NK, /µL | 88 (32-156) | 76 (34-109) | 0.337 |
| CD4+/ CD8 | 1.7 (0.8-2.7) | 1.9 (1.3-2.4) | 0.410 |

Values are presented as median (interquartile range). All P-values were evaluated by the chi-squared test, Fisher's exact test, Student's t test or Mann-Whitney's U test, as appropriate. *P < 0.05.

**Supplementary Figure S1. STROBE flow diagram of patient selection.** A total of 538 MDA5+DM patients were initially screened. After excluding patients with coexisting autoimmune diseases, malignancy, or incomplete data, 437 eligible patients remained. Among these, 50 patients had SPM. From the contemporaneous eligible patients without SPM, 100 were randomly selected at a 1:2 ratio to form the non-SPM control group. No matching on age or sex was performed. The final analysis included 50 SPM patients and 100 non-SPM patients. SPM, Spontaneous pneumomediastinum.
